# Supplementary material for: Self-attenuating adenovirus enables production of recombinant adeno-associated virus for high manufacturing yield without contamination
Source: Nat Commun. 2022 Mar 7;13:1182. doi: 10.1038/s41467-022-28738-2 (PMC8901928; doi:10.1038/s41467-022-28738-2)
Supplement: Supplementary file 1 — Supplementary Information [file 41467_2022_28738_MOESM1_ESM.pdf]

# Self-attenuating adenovirus enables propagation of recombinant adeno-associated virus for high manufacturing yield without contamination

**Weiheng Su[1,2], Maria I. Patrício[2], Margaret R. Duffy[1], Jakub M Krakowiak[2], Leonard W. Seymour[1] and Ryan Cawood[2]**

*[1]Department of Oncology, University of Oxford, Old Road Campus OX3 7DQ*

*[2]OXGENE Ltd, Oxford Science Park OX4 4GA*

## Supplementary Figures 1-9

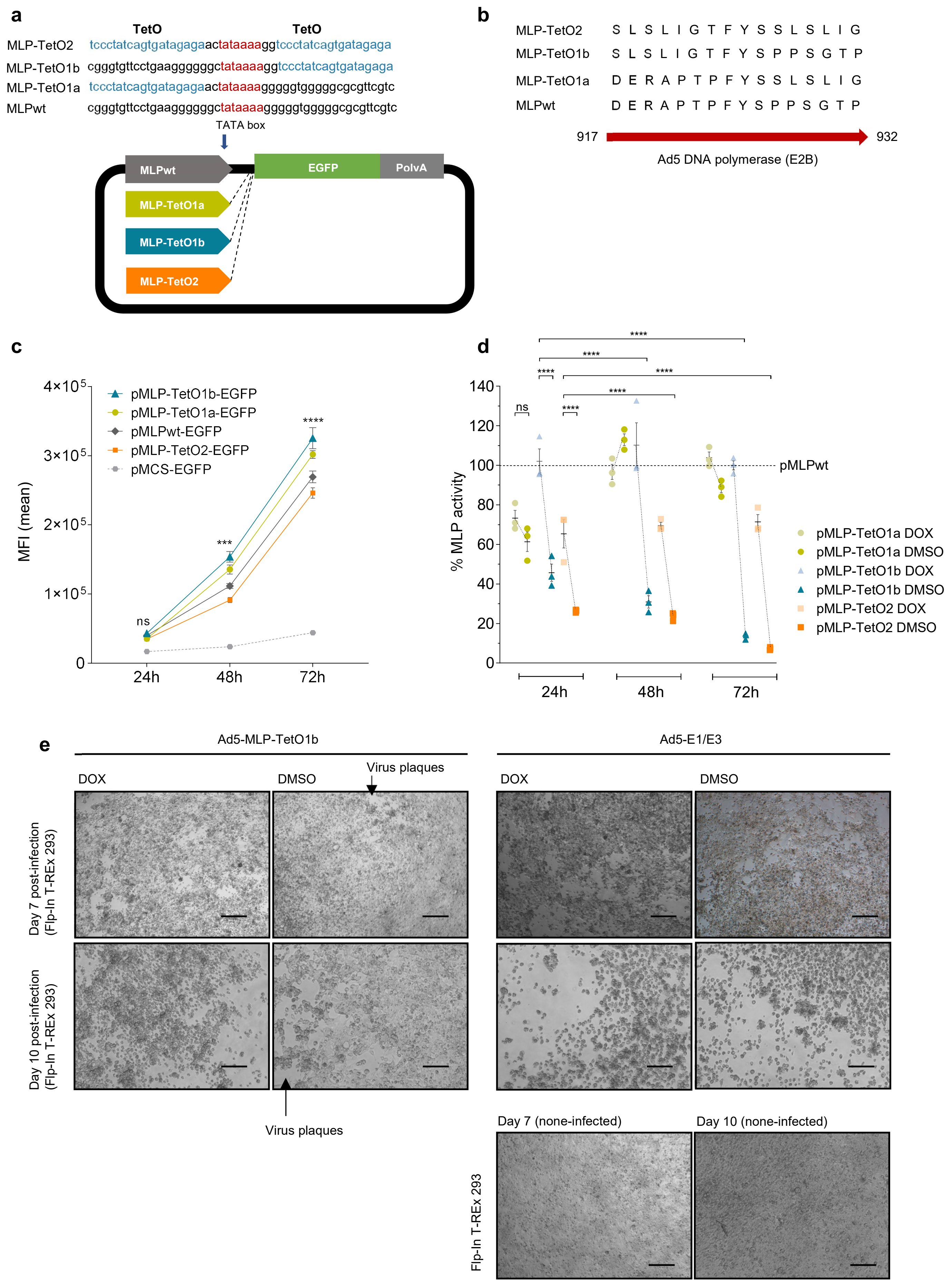
a
TetO
TetO
MLP-TetO2 tccctatcagtgatagagaactataaaaggtccctatcagtgatagaga
MLP-TetO1b cgggtgttcctgaaggggggctataaaaggtccctatcagtgatagaga
MLP-TetO1a tccctatcagtgatagagaactataaaagggggtgggggcgcgttcgtc
MLPwt cgggtgttcctgaaggggggctataaaagggggtgggggcgcgttcgtc
TATA box
MLPwt
EGFP
PolvA
MLP-TetO1a
MLP-TetO1b
MLP-TetO2
b
MLP-TetO2 S L S L I G T F Y S S L S L I G
MLP-TetO1b S L S L I G T F Y S P P S G T P
MLP-TetO1a D E R A P T P F Y S S L S L I G
MLPwt D E R A P T P F Y S P P S G T P
917
932
Ad5 DNA polymerase (E2B)
c
MFI (mean)
4×10^5
3×10^5
2×10^5
1×10^5
0
24h
48h
72h
ns
***
****
pMLP-TetO1b-EGFP
pMLP-TetO1a-EGFP
pMLPwt-EGFP
pMLP-TetO2-EGFP
pMCS-EGFP
d
% MLP activity
140
120
100
80
60
40
20
0
24h
48h
72h
ns
****
pMLPwt
pMLP-TetO1a DOX
pMLP-TetO1a DMSO
pMLP-TetO1b DOX
pMLP-TetO1b DMSO
pMLP-TetO2 DOX
pMLP-TetO2 DMSO
e
Ad5-MLP-TetO1b
Ad5-E1/E3
DOX
DMSO
Virus plaques
Day 7 post-infection (Flp-In T-REx 293)
Day 10 post-infection (Flp-In T-REx 293)
Day 7 (none-infected)
Day 10 (none-infected)
Flp-In T-REx 293

**Supplementary Figure 1:** (**a**) Schematic of Ad5 MLP expression plasmids and positioning of TetO sequences relative to the promoter TATA-box. (**b**) Resulting amino acid changes in the Ad5 DNA polymerase due to insertion of TetO sequences in the MLP. (**c**) Mean fluorescent intensity (MFI) of EGFP expressing HEK293 cells transfected with pMLP-TetO, pMLPwt or promoterless (pMCS) EGFP plasmids. Data is N=3 (mean±SD) of biological replicates. Analysed by two-way ANOVA followed by Bonferroni post hoc test comparing pMLP-TetO1b-EGFP versus pMLPwt-EGFP. ***$p < 0.0002$, ****$p \leq 0.0001$. (**d**) Transcriptional repression of MLP-TetO promoters by plasmid encoding TetR in HEK293 cells treated with doxycycline or DMSO. Data presented as percentage of MLP transcription activity, based on MFI of EGFP-positive cells, compared to wildtype pMLPwt promoter. Data normalised for background EGFP expression using pMCS-EGFP. Data is N=3 (mean±SEM) biological replicates. Analysed by two-way ANOVA with Bonferroni post hoc testing. ****$p \leq 0.0001$. (**e**) Flp-In T-REx 293 cells (stably expressing the TetR) were infected (MOI of 1) with Ad5-MLP-TetO1b or the control Ad5-E1/E3 and treated with doxycycline 0.2 μg/mL or DMSO. Cell monolayer imaged by brightfield microscopy at day 7 and day 10 post-infection. Virus plaques observed from Ad5-MLP-TetO1b (DMSO-group) are indicated. Data representative of N=3 biological replicate wells. Scale bar, 250 μm.

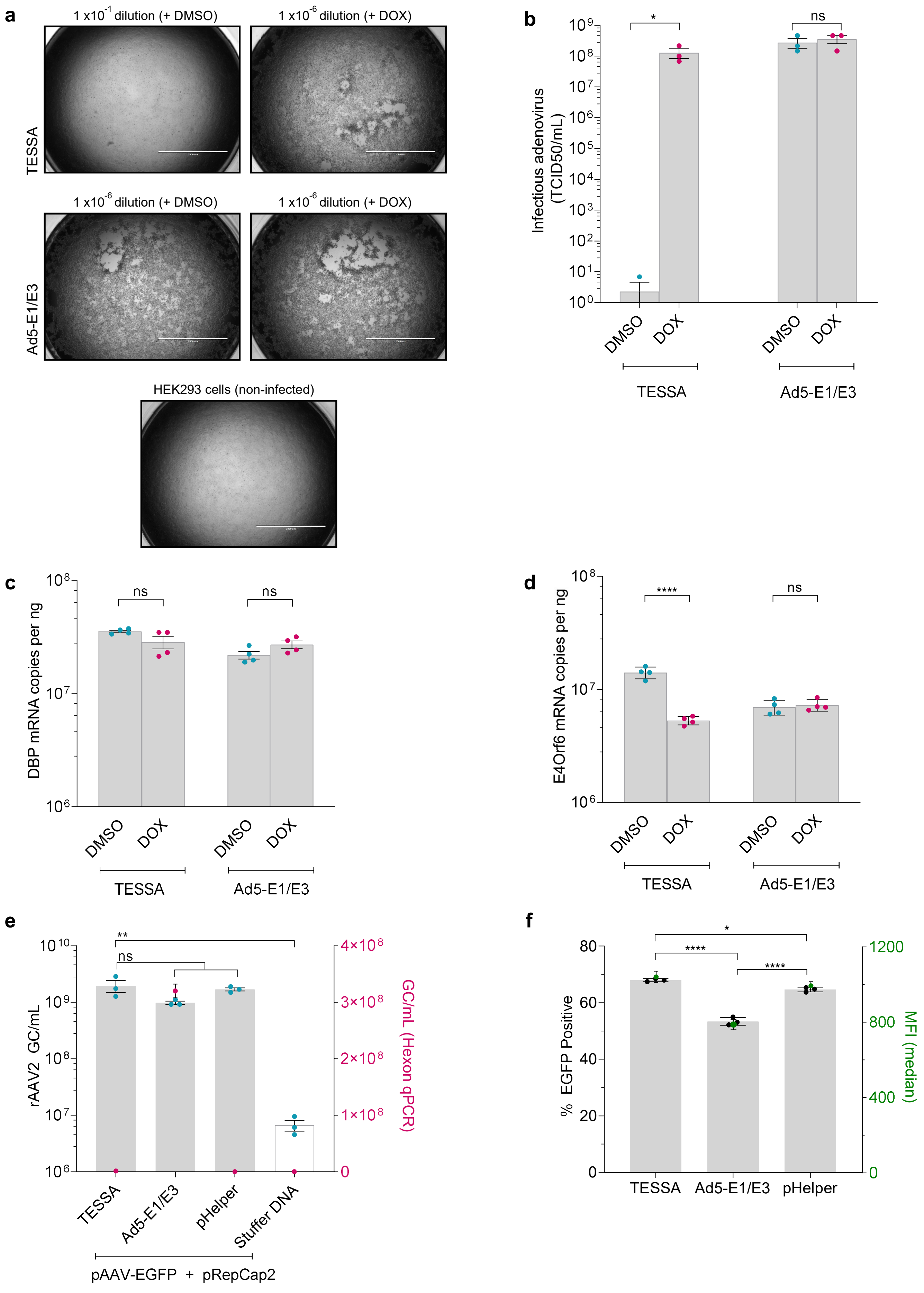
a
1 x10^-1 dilution (+ DMSO)
1 x10^-6 dilution (+ DOX)
TESSA
1 x10^-6 dilution (+ DMSO)
1 x10^-6 dilution (+ DOX)
Ad5-E1/E3
HEK293 cells (non-infected)
b
Infectious adenovirus (TCID50/mL)
10^0 10^1 10^2 10^3 10^4 10^5 10^6 10^7 10^8 10^9
*
ns
DMSO DOX DMSO DOX
TESSA Ad5-E1/E3
c
DBP mRNA copies per ng
10^6 10^7 10^8
ns ns
DMSO DOX DMSO DOX
TESSA Ad5-E1/E3
d
E4Orf6 mRNA copies per ng
10^6 10^7 10^8
**** ns
DMSO DOX DMSO DOX
TESSA Ad5-E1/E3
e
rAAV2 GC/mL
10^6 10^7 10^8 10^9 10^10
GC/mL (Hexon qPCR)
0 1×10^8 2×10^8 3×10^8 4×10^8
** ns
TESSA Ad5-E1/E3 pHelper Stuffer DNA
pAAV-EGFP + pRepCap2
f
% EGFP Positive
0 20 40 60 80
MFI (median)
0 400 800 1200
* **** ****
TESSA Ad5-E1/E3 pHelper

**Supplementary Figure 2:** (**a**) Representative brightfield images of HEK293 cells infected, at the indicated dilution, with crude preparation of TESSA and control Ad5 E1/E3-deleted (both doxycycline and DMSO group). Cells were cultured with doxycycline and imaged at day 9 post-infection. Scale bar, 2000 µm. Data representative of at least three independent experiments. (**b**) Quantification of contaminating adenovirus from HEK293 cells infected with TESSA compared to the Ad5-E1/E3 vector control using a TCID50 assay. HEK293 cells were inoculated with TESSA or Ad5 vectors at an MOI of 5 and cultured with DMSO or doxycycline 0.5 µg/mL. TCID50 assay carried out in HEK293 cells supplemented with doxycycline 0.5 µg/mL. Data is N=3 (mean±SEM) biological replicates. Statistical significance was calculated using an unpaired t-test (two-tailed). $*p = 0.0464$. Expression of (**c**) Ad5 E2A DNA binding protein (DBP) and (**d**) Ad5 E4Orf6 mRNA as determined by RT-qPCR from HEK293 cells infected with TESSA or Ad5-E1/E3 (used at an MOI of 10) and cultured with DMSO or doxycycline. Data is N=4 (mean±SEM) of biological replicates. (**e**) Production of rAAV2-EGFP from HEK293 cells transfected with pRepCap and pAAV, and infected with TESSA or the control Ad5-E1/E3 vector (used at an MOI of 10). Production yield was compared to transfection with pHelper (HF method). Cells transfected with stuffer DNA (pUC19 plasmid) to determine efficiency of the DNAse treatment is shown. Quantification of TESSA and Ad5-E1/E3 vectors by hexon-specific qPCR are shown on the right axis. Data is N=3 (mean±SEM) biological replicates. (**f**) Assessment of rAAV infectivity in HEK293 cells. Crude rAAV2-EGFP preparations were heat-treated at 60°C for 30 minutes to inactivate contaminating adenoviruses before infection of HEK293 cells at a normalised 100 GC/cell. EGFP expression was analysed by flow cytometry at 48 hpi and MFI (median) of EGFP positive cells are shown on the right axis. Data is N=3 (mean±SD) biological replicates. For panels **c-f**, statistical significance was determined using one-way ANOVA with Tukey's multiple comparisons. $*p = 0.0137$, $**p = 0.0022$, $****p \leq 0.0001$.

**a**

| Name | TCID50/mL | VP/mL | P/I ratio |
|---|---|---|---|
| Ad5-AAV | $7.1 \times 10^{11}$ (SD ± $8.2 \times 10^{10}$) | $3.1 \times 10^{12}$ (SD ± $1.6 \times 10^{11}$) | 4.37 |
| TESSA-AAV | $6.5 \times 10^{11}$ (SD ± $1.5 \times 10^{11}$) | $3.9 \times 10^{12}$ (SD ± $1.4 \times 10^{11}$) | 6.00 |

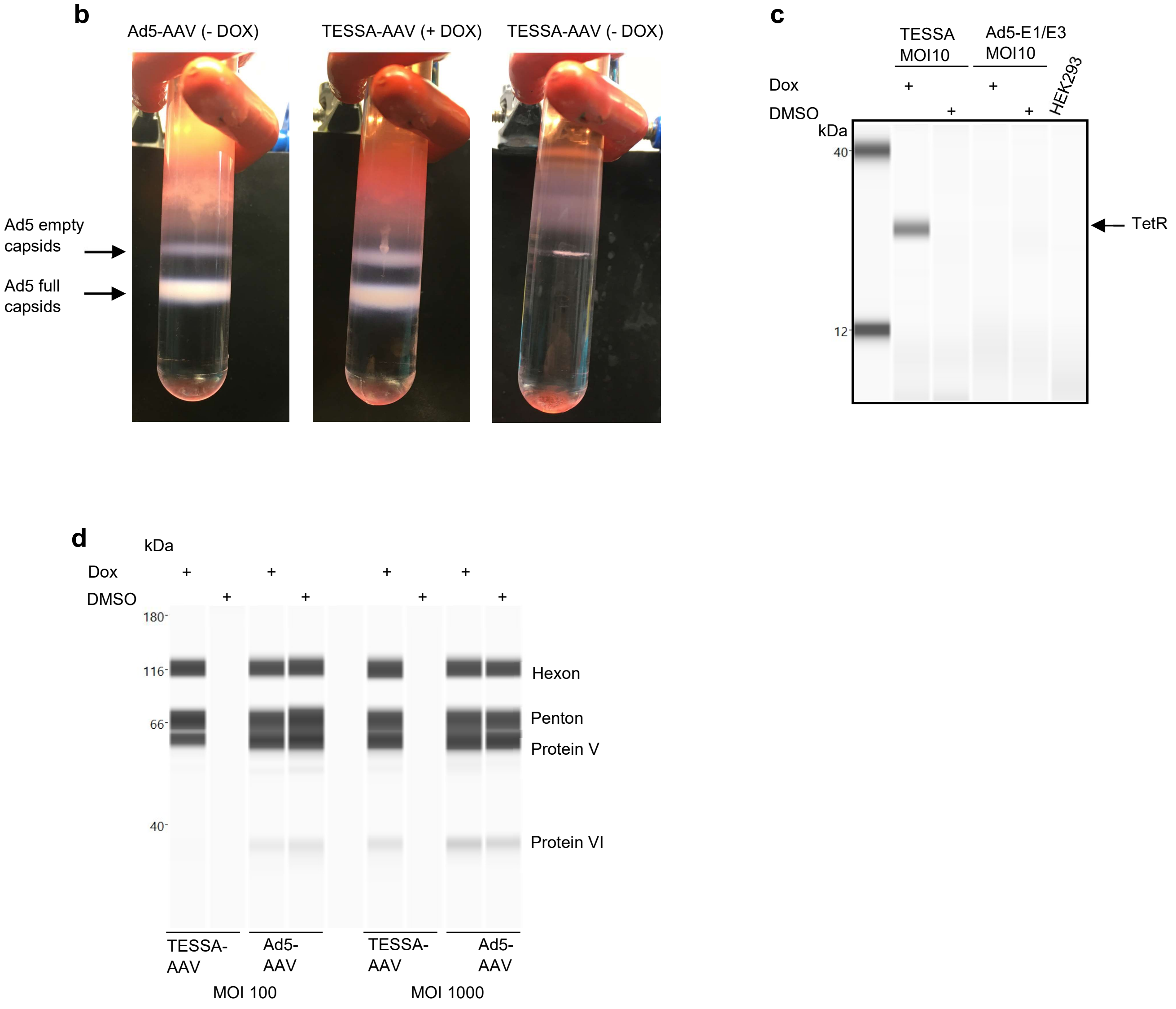

**Supplementary Figure 3:** (**a**) Comparison of infectious adenovirus (TCID50/mL) and viral particle (VP/mL) titres determined using a TCID50 and PicoGreen assay, respectively, from CsCl-purified TESSA-AAV and Ad5-AAV vector stocks. Data is N=3 (mean±SD) technical measurement (representative of at least three individual experiments). (**b**) HEK293 cells seeded in HYPERFlasks were infected (MOI of 5) with Ad5-AAV or TESSA-AAV (with and without doxycycline 0.5 μg/mL). Adenovirus particles were harvested at 72 hours post-infection and purified by CsCl density gradient. Representative images after one round of ultracentrifugation are shown. The lower larger band corresponds to intact viral particles containing the virus genome and the upper smaller band formed by empty particles are indicated. Data representative of two independent replicates. (**c**) Western blot detection of TetR proteins expressed from TESSA compared to control Ad5-E1/E3 in HEK293 cells treated with DMSO or doxycycline. (**d**) Western blot detection of adenovirus structural proteins expressed from TESSA-AAV in HEK293 cells treated with DMSO or doxycycline. Samples from cells infected with the control Ad5-AAV are also shown. Data from panels **c**-**d** is representative of at least three independent experiments.

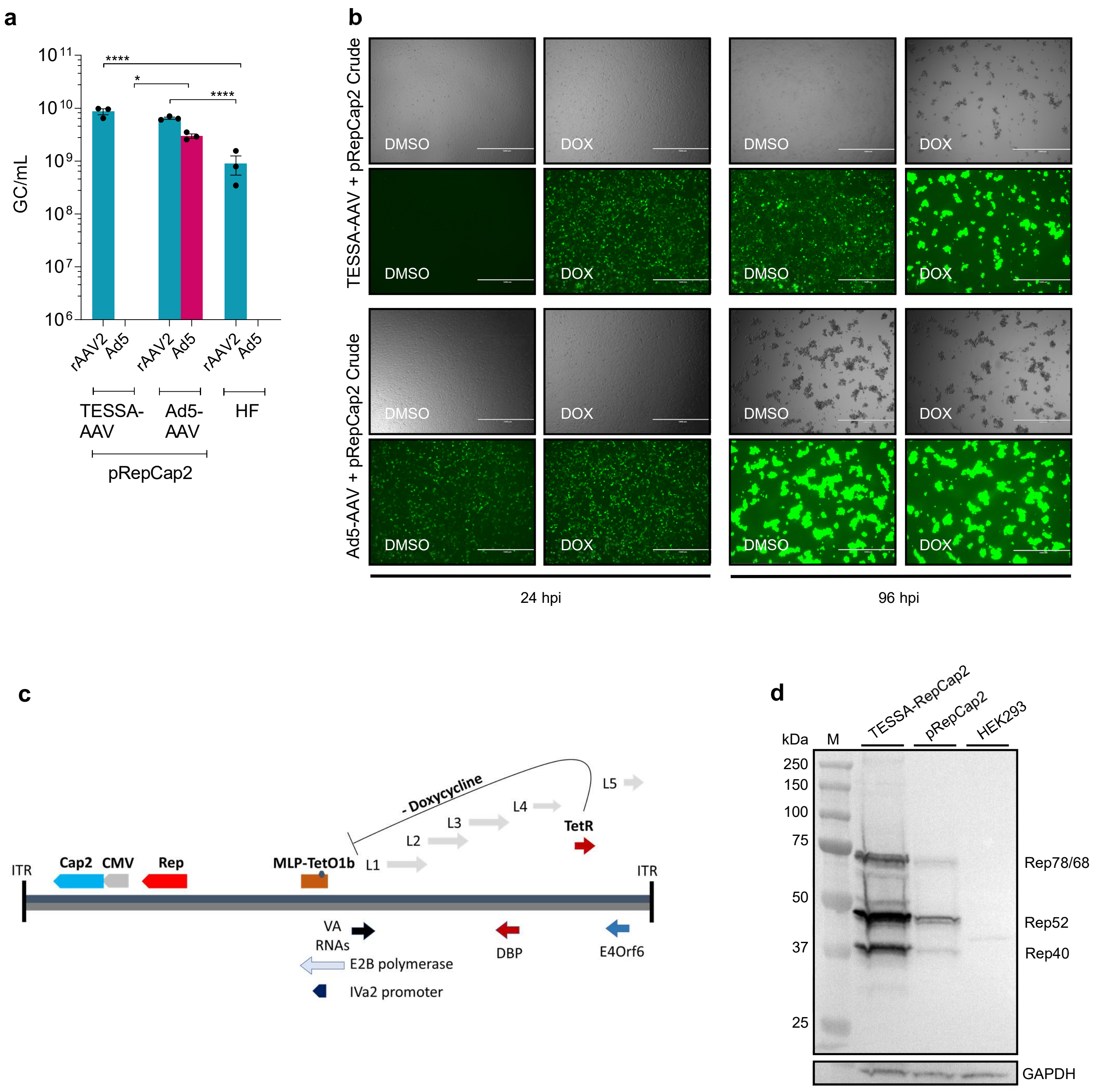
a
GC/mL
rAAV2
Ad5
TESSA-AAV
Ad5-AAV
HF
pRepCap2
b
TESSA-AAV + pRepCap2 Crude
Ad5-AAV + pRepCap2 Crude
DMSO
DOX
24 hpi
96 hpi
c
ITR
Cap2
CMV
Rep
MLP-TetO1b
L1
L2
L3
L4
L5
TetR
- Doxycycline
VA RNAs
E2B polymerase
IVa2 promoter
DBP
E4Orf6
d
kDa
M
TESSA-RepCap2
pRepCap2
HEK293
250
150
100
75
50
37
25
Rep78/68
Rep52
Rep40
GAPDH

**Supplementary Figure 4**: (**a**) rAAV2 production using TESSA-AAV or Ad5-AAV compared to HF method. HEK293 cells were either transfected with pRepCap2 prior to infection (MOI of 500) with Ad5-AAV or TESSA-AAV, or transfected with the HF plasmids. Viruses were extracted 96 hpt and encapsulated adenovirus and rAAV2 quantified by hexon (specific for adenovirus) and EGFP-qPCR (detecting both adenovirus and AAV). rAAV yield was calculated by subtracting the hexon signal from the EGFP signal. Data is N=3 (mean±SEM) biological replicates. Statistical significance was determined using one-way ANOVA with Tukey's multiple comparisons. *$p$ = 0.0110, ****$p \leq 0.0001$. (**b**) HEK293 cells infected (1 in 100 dilutions) with crude rAAV2-EGFP produced from TESSA-AAV or Ad5-AAV with DMSO or doxycycline supplement. Cells were supplemented with doxycycline and imaged by brightfield and fluorescent microscopy. Scale bar, 1000 µm. Data representative of N=3 biological replicates. (**c**) Schematic representation of TESSA encoding AAV2 Cap under control of a CMV promoter and the AAV2 Rep. (**d**) Western blot analysis of AAV Rep expressed from TESSA-RepCap2 (used at 25 GC/cell) in HEK293 cells compared to transfection with plasmid pRepCap2. Cells were harvested at 24 hpt. Equal amounts (25 µL) of cellular lysates were analysed by western blot using anti-Rep and anti-GAPDH antibodies as a loading control. Data representative of at least three independent experiments.

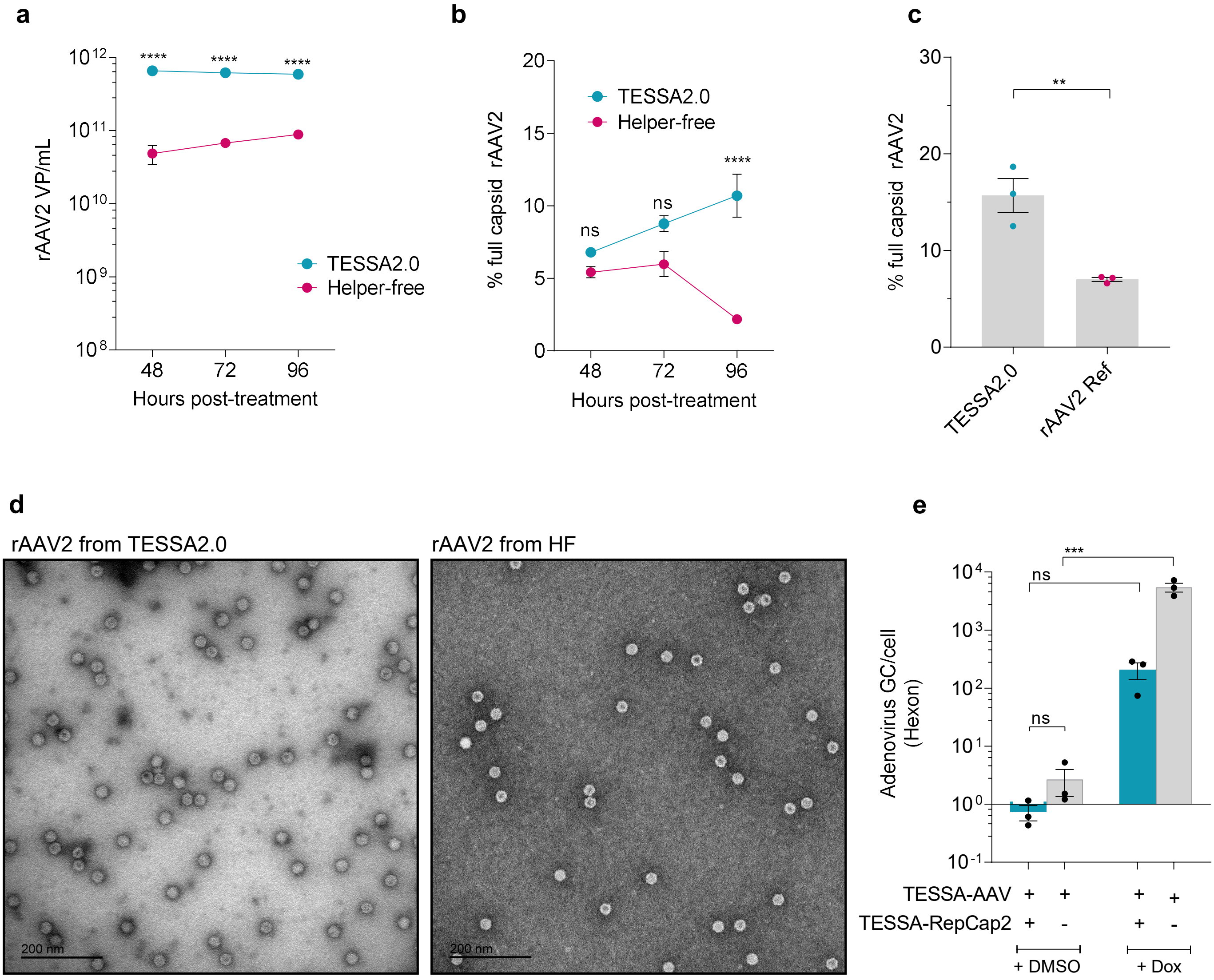

a
rAAV2 VP/mL
Hours post-treatment
TESSA2.0
Helper-free
****
b
% full capsid rAAV2
Hours post-treatment
TESSA2.0
Helper-free
ns
****
c
% full capsid rAAV2
TESSA2.0
rAAV2 Ref
**
d
rAAV2 from TESSA2.0
rAAV2 from HF
200 nm
e
Adenovirus GC/cell (Hexon)
TESSA-AAV
TESSA-RepCap2
+ DMSO
+ Dox
ns
***

**Supplementary Figure 5**: (**a**) Production or rAAV2-EGFP in HEK293 cells using the TESSA2.0 vectors (TESSA-AAV and TESSA-RepCap2 used at an MOI of 25) versus transfection with the HF plasmids. Assembled viral particles (VP) quantified by AAV2-specific ELISA. (**b**) Proportion of encapsulated rAAV2 genomes (% full) determined following quantification of assembled rAAV2 particles using ELISA and compared against the qPCR titre. Data is N=3 (mean±SD) biological replicates. Analysed by two-way ANOVA followed by Bonferroni post hoc test comparing TESSA2.0 versus Helper-free. ****$p \leq 0.0001$. (**c**) Proportion of encapsulated rAAV2 genomes (% full) from purified rAAV2-EGFP stock derived from TESSA2.0 compared to a rAAV2-EGFP reference control (Vector Biolabs). Results were determined following quantification of assembled rAAV2 particles using ELISA and compared against the qPCR titre. Data is N=3 (mean±SEM) biological replicates. **$p = 0.00821$ (two-tailed unpaired t-test). (**d**) Transmission electron microscopy imaging of negatively stained (2% uranyl acetate) purified rAAV2-EGFP produced via TESSA2.0 or HF method. Scale bar, 200 nm. Data representative of at least three independent experiments. **(e)** Assessment of adenovirus levels from the TESSA2.0 system. HEK293 cells were infected with two TESSA vectors (TESSA-AAV and TESSA-RepCap2) or just TESSA-AAV (MOI of 25). Cells were cultured in the presence of DMSO or doxycycline (0.5 µg/mL) for 96 hours and DNAse-resistant adenovirus genomes were quantified by qPCR against Ad5 hexon. Data is N=3 (mean±SEM) of biological replicates. Statistical significance was determined using one-way ANOVA with Tukey's multiple comparisons. ***$p = 0.0002$.

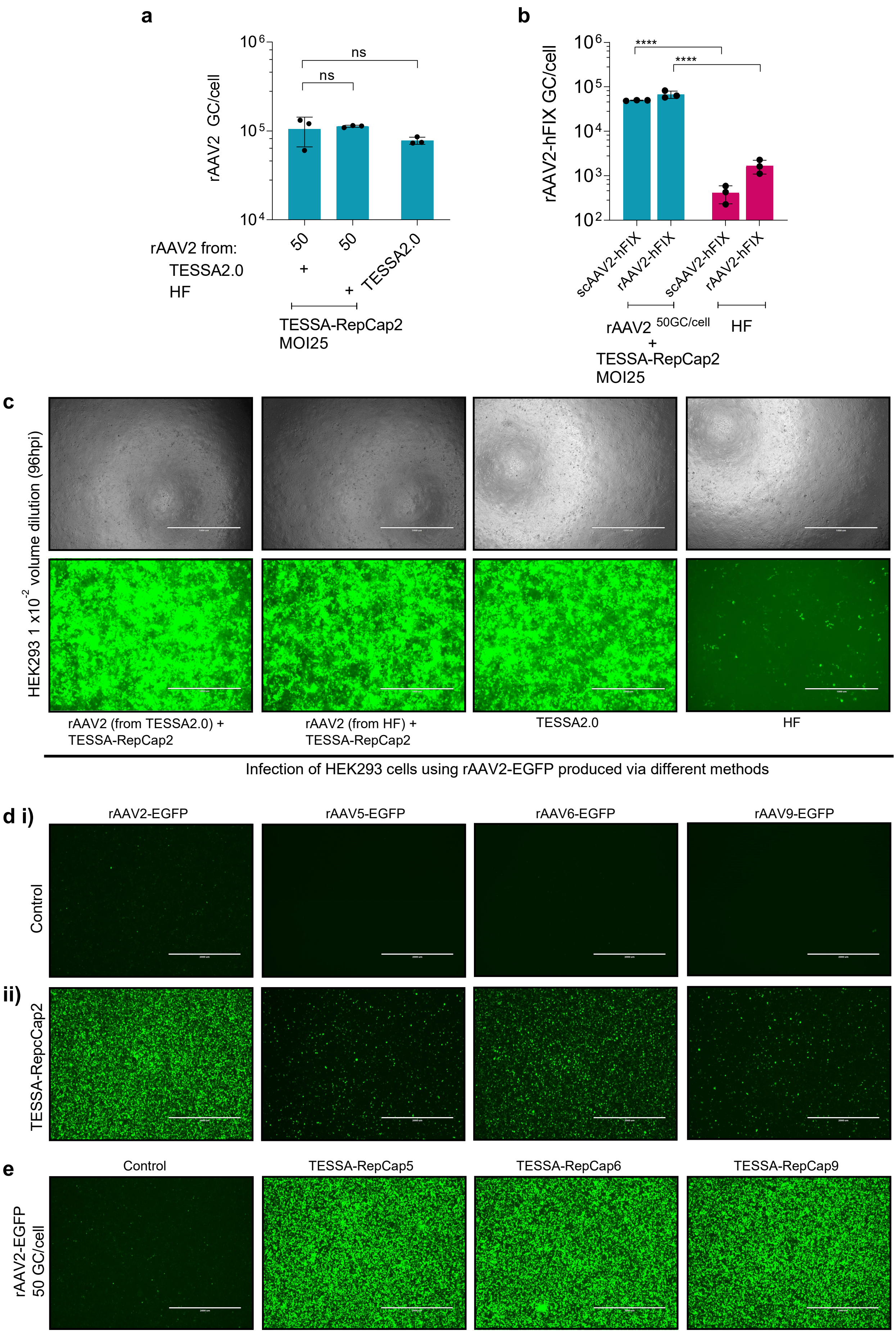

a
rAAV2 GC/cell
10^6
10^5
10^4
ns
ns
rAAV2 from:
TESSA2.0
HF
50
50
TESSA2.0
+
+
TESSA-RepCap2
MOI25
b
rAAV2-hFIX GC/cell
10^6
10^5
10^4
10^3
10^2
****
****
scAAV2-hFIX
rAAV2-hFIX
scAAV2-hFIX
rAAV2-hFIX
rAAV2 50GC/cell
+
TESSA-RepCap2
MOI25
HF
c
HEK293 1 x10^-2 volume dilution (96hpi)
rAAV2 (from TESSA2.0) +
TESSA-RepCap2
rAAV2 (from HF) +
TESSA-RepCap2
TESSA2.0
HF
Infection of HEK293 cells using rAAV2-EGFP produced via different methods
d i)
rAAV2-EGFP
rAAV5-EGFP
rAAV6-EGFP
rAAV9-EGFP
Control
ii)
TESSA-RepcCap2
e
Control
TESSA-RepCap5
TESSA-RepCap6
TESSA-RepCap9
rAAV2-EGFP
50 GC/cell

**Supplementary Figure 6:** (**a**) Production of rAAV2-EGFP via propagation using TESSA-RepCap2. HEK293 cells were co-infected using rAAV2 produced via HF method or TESSA2.0 (50 GC/cell) with TESSA-RepCap2 (MOI of 25). Production yield compared to TESSA2.0 (used at MOI of 25) approach. DNAse-resistant particles quantified by EGFP-specific qPCR at 96 hpi. Data presented as N=3 (mean±SD) biological replicates (ns, one-way ANOVA with Tukey's multiple comparisons). (**b**) Production yield of rAAV2 or self-complementing (sc) rAAV2 vector encoding human FIX via propagation using TESSA-RepCap2. Cells were co-infected with rAAV2-hFIX or scAAV2-hFIX (produced by HF method) at 50 GC/cell with TESSA-RepCap2 (MOI of 25) or subjected to plasmid HF transfection. DNAse-resistant particles quantified by hFIX-specific qPCR at 96 hpi. Data presented as N=3 (mean±SD) biological replicates. Statistical significance was determined using one-way ANOVA with Tukey's multiple comparisons. ****$p \leq 0.0001$. (**c**) Representative brightfield and fluorescent images of HEK293 cells transduced (at a 1:100 volume dilution) with crude rAAV2-EGFP preparations derived from the different production approaches. Cells imaged at 96 hpi. Scale bar, 1000 μm. (**d**) Representative fluorescence images of HEK293 cells transduced with (**i**) rAAV2-, rAAV5-, rAAV6- and rAAV9-EGFP vectors at 50 GC/cell (HF-derived, control), (**ii**) and in the presence of TESSA-RepCap2 (MOI of 25). Cells were imaged at 48 hpi. Scale bar, 2000 μm. (**e**) Representative fluorescence images of HEK293 cells infected (at 50 GC/cell) with rAAV2-EGFP only (HF-derived, control) or co-infected with TESSA-RepCap5 (at 100 GC/cell), TESSA-RepCap6 (at 100 GC/cell), or TESSA-RepCap9 (at 75 GC/cell). Cells were imaged at 48 hpi. Scale bar, 2000 μm. For panels **c**-**e**, data representative of at least three independent experiments.

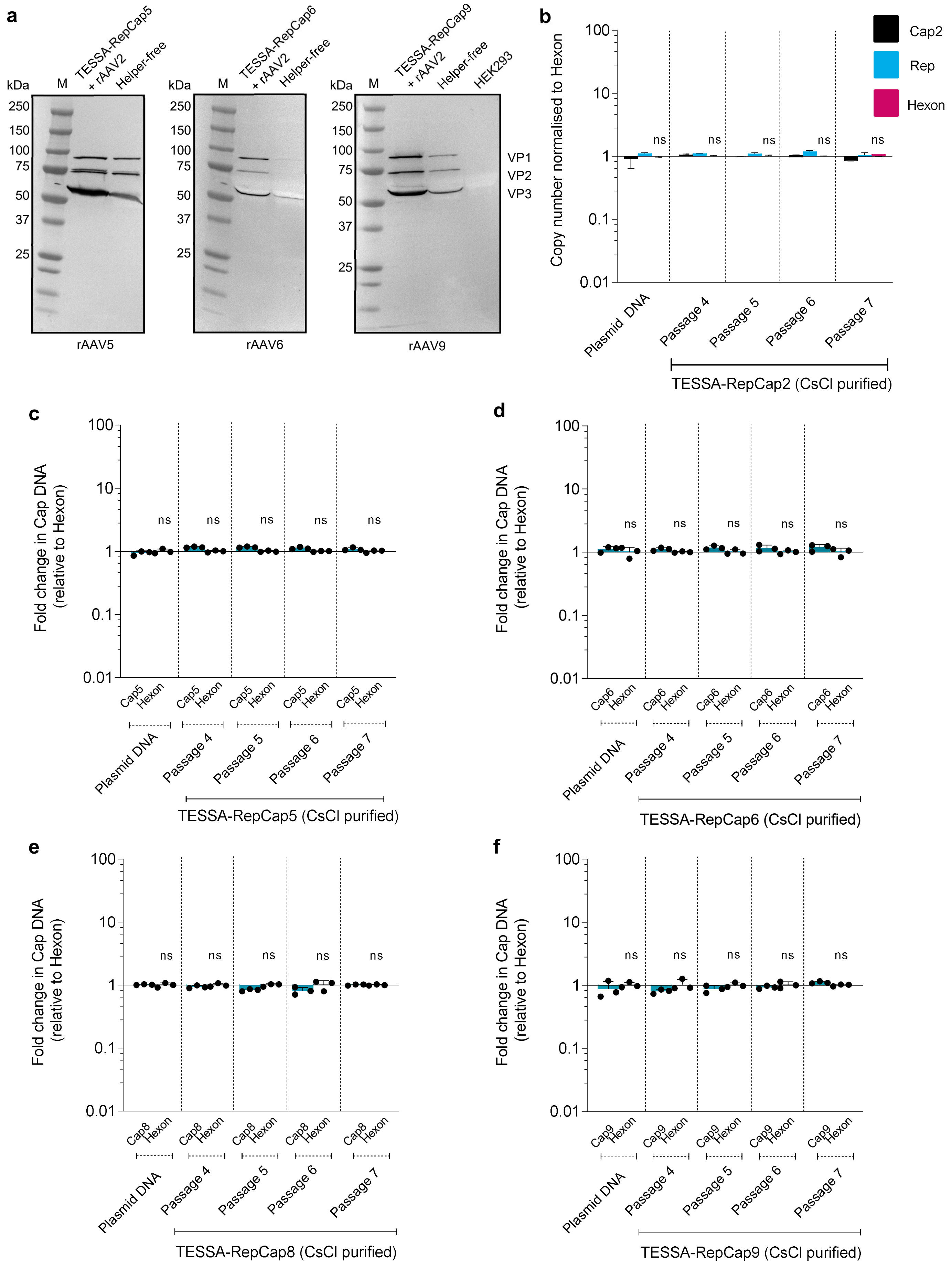
a
kDa
M
TESSA-RepCap5 + rAAV2
Helper-free
250
150
100
75
50
37
25
rAAV5
TESSA-RepCap6 + rAAV2
rAAV6
TESSA-RepCap9 + rAAV2
HEK293
rAAV9
VP1
VP2
VP3
b
Copy number normalised to Hexon
100
10
1
0.1
0.01
ns
Cap2
Rep
Hexon
Plasmid DNA
Passage 4
Passage 5
Passage 6
Passage 7
TESSA-RepCap2 (CsCl purified)
c
Fold change in Cap DNA (relative to Hexon)
Cap5
Hexon
TESSA-RepCap5 (CsCl purified)
d
Cap6
TESSA-RepCap6 (CsCl purified)
e
Cap8
TESSA-RepCap8 (CsCl purified)
f
Cap9
TESSA-RepCap9 (CsCl purified)

**Supplementary Figure 7:** (**a**) Western blot analysis of rAAV5-EGFP, rAAV6-EGFP and rAAV9-EGFP capsid proteins from particles produced using rAAV2 capsid-exchange or helper-free method. Equal amounts of crude preparations (25 µL) containing rAAV5, rAAV6 and rAAV9-EGFP produced via helper-free method or co-infection of rAAV2-EGFP (50 GC/cell, HF-derived) with TESSA-RepCap5 (100 GC/cell), TESSA-RepCap6 (100 GC/cell), TESSA-RepCap9 (75 GC/cell) were probed with anti-AAV VP1/2/3 antibody for western blot detection. Data representative of at least three independent experiments. (**b**) Assessment of TESSA-RepCap2 genetic stability at serial passages by qPCR analysis. DNA extracted from purified TESSA-RepCap2 vector at the indicated passages were quantified by qPCR against AAV2 rep and cap, and Ad5 hexon. Data shows the copy number of AAV2 rep and cap genes relative to Ad5 hexon and compared to DNA plasmid encoding TESSA-RepCap2. Genetic stability assessment of (**c**) TESSA-RepCap5, (**d**) TESSA-RepCap6, (**e**) TESSA-RepCap8, and (**f**) TESSA-RepCap9 are shown. In each assessment, data shows the copy number of cap genes relative to Ad5 hexon, and compared to the DNA plasmid control. For panels **b-f**, data presented as N=3 (mean±SD) technical replicates (ns, analysed by two-way ANOVA with Bonferroni post hoc testing comparing Ad5 hexon to AAV rep and cap).

**a**

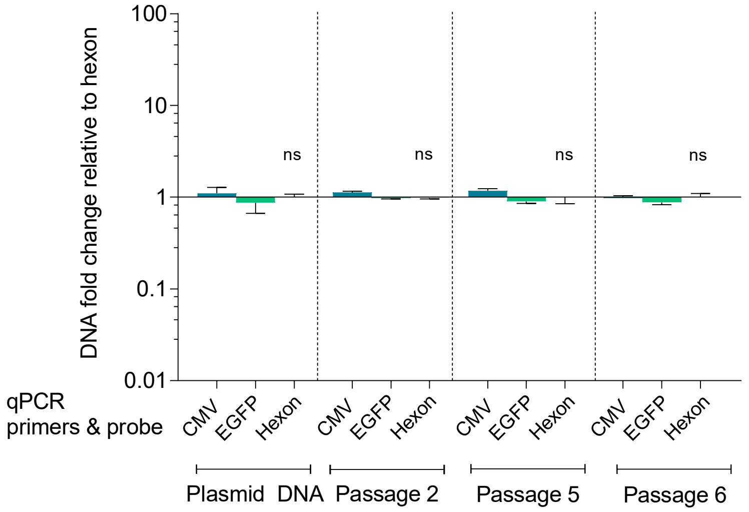


**b**

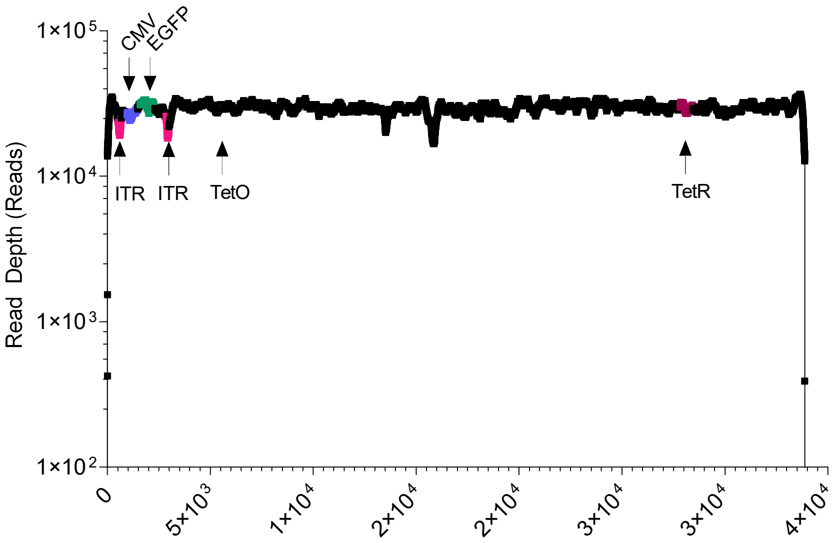


**c**

Wells positive for adenovirus plaques
(40 replicated wells per test dilution)

| Dilution factor | rAAV2-EGFP via TESSA2.0 (crude) | rAAV2-EGFP via TESSA2.0 (AAVX purified) | rAAV2-EGFP via passage (crude) | rAAV2-EGFP via passage (AAVX purified) |
|---|---|---|---|---|
| $1 \times 10^{-2}$ | NA | 0 / 40 | NA | 0 / 40 |
| $1 \times 10^{-3}$ | 10 / 40 | 0 / 40 | 0 / 40 | 0 / 40 |
| $1 \times 10^{-4}$ | 1 / 40 | 0 / 40 | 0 / 40 | 0 / 40 |
| $1 \times 10^{-5}$ | 0 / 40 | 0 / 40 | 0 / 40 | 0 / 40 |

**Supplementary Figure 8:** (**a**) Assessment of TESSA-AAV genetic stability at serial passages by qPCR analysis. DNA extracted from purified TESSA-AAV vectors at the indicated serial passages were quantified by qPCR against the CMV promoter, EGFP, and Ad5 hexon. Data shows the copy number of CMV and EGFP DNA relative to Ad5 hexon and compared to DNA plasmid encoding TESSA-AAV. Data presented as N=3 (mean±SD) technical replicates (ns, analysed by two-way ANOVA with Bonferroni post hoc testing). (**b**) Analysis of TESSA-AAV via NGS Illumina HiSeq 2x250 bp run. TESSA-AAV (1 x$10^{11}$ vector genomes) were extracted from CsCl-purified particles and used for sequencing. Sequencing reads were aligned to the reference sequence and sequencing depth was determined by SAMtools depth. 98% out of 5.1 million reads aligned to the reference sequence. Base position and sequencing depth of the AAV ITRs, transgene expression cassette, TetO, and TetR coding sequences within TESSA-AAV are highlighted. (**c**) Assessment of adenovirus contamination in crude and purified (via AAVX Affinity Resin) rAAV2 preparations derived from TESSA2.0, or via passage of rAAV2 (used at 50 GC/cell, HF-derived) using TESSA RepCap2 at an MOI of 25. rAAV2 were produced in 1L bioreactor culture of suspension HEK293 cells and infectious adenovirus quantified using a TCID50 assay in HEK293 cells (supplemented with doxycycline 0.5 μg/mL). Forty replicate tests of each dilution were carried out. Data representative of two independent experiments.

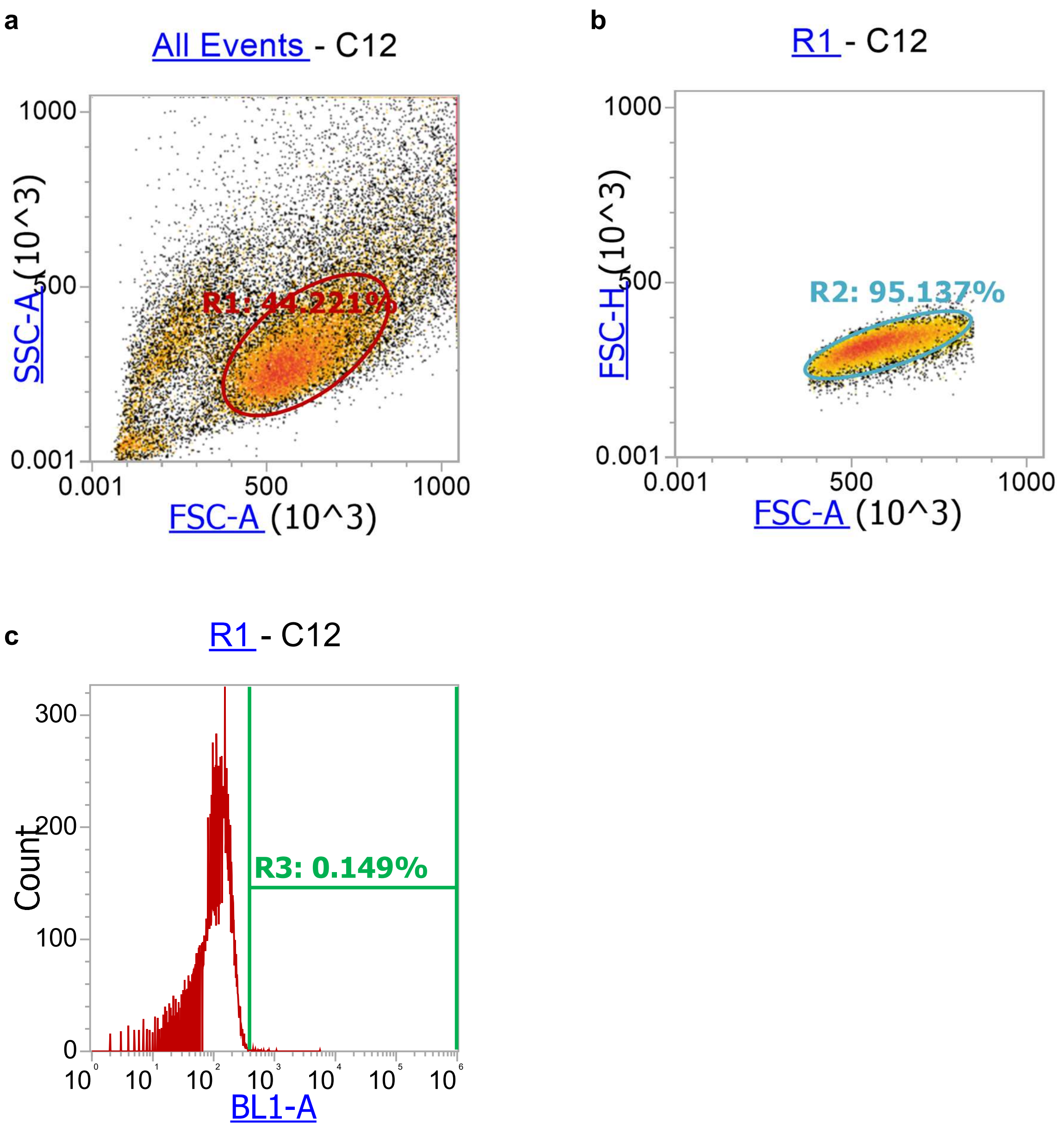


**Supplementary Figure 9:** Flow cytometry (Attune NxT software v3.1) gating strategy for defining the EGFP negative population of HEK293 cells. (**a**) SSC-FSC plot selecting live cell population. (**b**) FSC-H and FSC-A gate for exclusion of doublets. (**c**) BL1-A channel gating to measure EGFP expression.
