# Supplementary material for: Self-attenuating adenovirus enables production of recombinant adeno-associated virus for high manufacturing yield without contamination
Source: Nat Commun. 2022 Mar 7;13:1182. doi: 10.1038/s41467-022-28738-2 (PMC8901928; doi:10.1038/s41467-022-28738-2)
Supplement: Supplementary file 2 — Reporting Summary [file 41467_2022_28738_MOESM2_ESM.pdf]

nature research

Corresponding author(s): Leonard W. Seymour, Weiheng Su, Ryan Cawood

Last updated by author(s): 19/01/2022

Reporting Summary

Nature Research wishes to improve the reproducibility of the work that we publish. This form provides structure for consistency and transparency in reporting. For further information on Nature Research policies, see our Editorial Policies and the Editorial Policy Checklist.

Statistics

For all statistical analyses, confirm that the following items are present in the figure legend, table legend, main text, or Methods section.

n/a	Confirmed	
☐	☒	The exact sample size (*n*) for each experimental group/condition, given as a discrete number and unit of measurement
☐	☒	A statement on whether measurements were taken from distinct samples or whether the same sample was measured repeatedly
☐	☒	The statistical test(s) used AND whether they are one- or two-sided<br>*Only common tests should be described solely by name; describe more complex techniques in the Methods section.*
☒	☐	A description of all covariates tested
☐	☒	A description of any assumptions or corrections, such as tests of normality and adjustment for multiple comparisons
☐	☒	A full description of the statistical parameters including central tendency (e.g. means) or other basic estimates (e.g. regression coefficient) AND variation (e.g. standard deviation) or associated estimates of uncertainty (e.g. confidence intervals)
☐	☒	For null hypothesis testing, the test statistic (e.g. *F*, *t*, *r*) with confidence intervals, effect sizes, degrees of freedom and *P* value noted<br>*Give P values as exact values whenever suitable.*
☒	☐	For Bayesian analysis, information on the choice of priors and Markov chain Monte Carlo settings
☒	☐	For hierarchical and complex designs, identification of the appropriate level for tests and full reporting of outcomes
☒	☐	Estimates of effect sizes (e.g. Cohen's *d*, Pearson's *r*), indicating how they were calculated

Our web collection on statistics for biologists contains articles on many of the points above.

Software and code

Policy information about availability of computer code

Data collection	Microscopy: EVOS FL imaging system (ThermoFisher)<br>qPCR: StepOnePlus™ Real-Time PCR System (StepOne™ Software v2.3, Applied Biosystems)<br>Western Blot: Wes automated system (Compass for SW v4.1.0, ProteinSimple)<br>Flow cytometry: Attune NxT flow cytometer (Attune NxT software v3.1, ThermoFisher), Accuri C6 flow cytometer (BD Accuri C6 Software v227.4, BD Biosciences)<br>ELISA: FLUOstar Omega (MARS Data Analysis Software v5.70 , BMG Labtech)
Data analysis	qPCR, Flow cytometry and ELISA data analysis carried out using Microsoft Excel (Version 2102, Microsoft) and GraphPad PRISM (Version 8, GraphPad software, Inc). Western bot data analysis carried out using Compass for SW (version 4.1.0, ProteinSimple). Analysis of NGS (Illumina) sequencing reads were carried out using Integrative Genomics Viewer (IGV v2.11.9, Broad Institute), SAMtools (v1.14, Genome Research Limited) and BCFtools (v1.14, Genome Research Limited).

For manuscripts utilizing custom algorithms or software that are central to the research but not yet described in published literature, software must be made available to editors and reviewers. We strongly encourage code deposition in a community repository (e.g. GitHub). See the Nature Research guidelines for submitting code & software for further information.

Data

Policy information about availability of data

All manuscripts must include a data availability statement. This statement should provide the following information, where applicable:

- Accession codes, unique identifiers, or web links for publicly available datasets
- A list of figures that have associated raw data
- A description of any restrictions on data availability

All data generated throughout this study are included in this article and its Supplementary information files. Vector sequences have been made publicly available through published patent (WO/2019/020992). Plasmid DNA and viral vectors generated during the current study are available from the corresponding author on reasonable request.

Field-specific reporting

Please select the one below that is the best fit for your research. If you are not sure, read the appropriate sections before making your selection.

[x] Life sciences   [ ] Behavioural & social sciences   [ ] Ecological, evolutionary & environmental sciences

For a reference copy of the document with all sections, see nature.com/documents/nr-reporting-summary-flat.pdf

Life sciences study design

All studies must disclose on these points even when the disclosure is negative.

Sample size	For individual analyses n is either 3 or 4, selected on the basis that the variability between estimates is sufficiently small to provide significant differences between test samples in these studies.
Data exclusions	No data were excluded from the analyses
Replication	Information on statistics and reproducibility is included at the figure legend of each dataset. All results were repeated at least twice independently with similar results.
Randomization	Randomization was carried out as there were no group selection or in vivo animal assessment associated with this study. Viral stocks and cells used in this study were of sufficient high concentration and quantity to ensure randomization of infection events. All data were uniformly collected between the control and assessment groups.
Blinding	There was no group selection or in vivo animal assessment associated with this study, and therefore blinding is not applicable. Data analysis from this study was strictly quantitative and with objective outcomes.

Reporting for specific materials, systems and methods

We require information from authors about some types of materials, experimental systems and methods used in many studies. Here, indicate whether each material, system or method listed is relevant to your study. If you are not sure if a list item applies to your research, read the appropriate section before selecting a response.

Materials & experimental systems

n/a	Involved in the study	
[ ]	[x]	Antibodies
[ ]	[x]	Eukaryotic cell lines
[x]	[ ]	Palaeontology and archaeology
[x]	[ ]	Animals and other organisms
[x]	[ ]	Human research participants
[x]	[ ]	Clinical data
[x]	[ ]	Dual use research of concern

Methods

n/a	Involved in the study	
[x]	[ ]	ChIP-seq
[ ]	[x]	Flow cytometry
[x]	[ ]	MRI-based neuroimaging

Antibodies

Antibodies used	Anti adenovirus type 5 (ab6982, Abcam, Cambridge, UK)<br>Anti-TetR antibody (ab25845, Abcam, Cambridge, UK)<br>Anti-AAV2 Replicase mouse monoclonal, clone 303.9 (61069, Lot: 911110-02,Progen, Heidelberg, Germany)<br>Anti-AAV VP1/VP2/VP3 mouse monoclonal, clone B1 (65158, Lot: 712151-02, Progen, Heidelberg, Germany)<br>Anti-GAPDH antibody (2118, Cell Signaling Technology, MA, USA)

Secondary donkey anti-mouse, HRP (A16011, Lot: GR3313983-5, ThermoFisher, MA, USA)
Secondary goat anti-rabbit, HRP (31460, Lot: GR3259646-7, ThermoFisher, MA, USA)

Validation

All antibodies were purchased from commercial vendors and have been validated by the manufacturers on their official websites.

Anti adenovirus type 5 (ab6982, Abcam, Cambridge, UK) - validated for western blot by manufacturer. Reacts for Tissue, cells or virus corresponding to Adenovirus Type 5 and Purified Adenovirus type 5.
Anti-TetR antibody (ab25845, Abcam, Cambridge, UK) - validated for western blot by manufacturer. Reactivity is species independent. Detects Tet repressor protein.
Anti-AAV2 Replicase (61069, Lot: 911110-02,Progen, Heidelberg, Germany) - validated for western blot by manufacturer. Reacts with AAV2 Rep40, Rep52, Rep68 and Rep78.
Anti-AAV VP1/VP2/VP3 (65158, Lot: 712151-02, Progen, Heidelberg, Germany) - validated for western blot by manufacturer. Reacts with VP1, VP2, VP3 proteins of AAV1, 2, 3, 5, 6, 7, 8, 9, rh10, DJ.
Anti-GAPDH antibody (2118, Cell Signaling Technology, MA, USA) - validated for western blot by manufacturer. Reactivity with Human, Mouse, Rat, Monkey, Bovine, Pig for endogenous levels of total GAPDH protein.

Eukaryotic cell lines

Policy information about cell lines

Cell line source(s)	HEK293 (Cell Biolabs, USA); Flp-In T-REx 293 cells (ThermoFisher Scientific, USA); HeLa RC32 and U-87 MG cells (ATCC, USA); Suspension HEK293 cells (OXGENE, UK)
Authentication	Cell lines were authenticated at source and regularly validate by microsatellite analysis
Mycoplasma contamination	Cell lines were routinely tested for mycoplasma contamination and found to be negative
Commonly misidentified lines (See ICLAC register)	No commonly misidentified cell lines were used

Flow Cytometry

Plots

Confirm that:

- [x] The axis labels state the marker and fluorochrome used (e.g. CD4-FITC).
- [x] The axis scales are clearly visible. Include numbers along axes only for bottom left plot of group (a 'group' is an analysis of identical markers).
- [x] All plots are contour plots with outliers or pseudocolor plots.
- [x] A numerical value for number of cells or percentage (with statistics) is provided.

Methodology

Sample preparation	samples are prepared using established protocols
Instrument	Attune NxT Flow Cytometer (ThermoFisher, MA, USA) or accuri C6 flow cytometer (BD Biosciences, NJ, USA)
Software	Attune NxT and Accuri C6 software were used for FACS analysis
Cell population abundance	At least 10,000 cells were analysed in each cell fraction reported
Gating strategy	Cells were gated using FSC/SSC to exclude dead cells and then fluorescence baselines were set to exclude 99% of control cells

- [x] Tick this box to confirm that a figure exemplifying the gating strategy is provided in the Supplementary Information.
